# Baseline interleukin-6 is a prognostic factor for patients with metastatic breast cancer treated with eribulin

**DOI:** 10.1007/s10549-023-07086-9

**Published:** 2023-09-21

**Authors:** Ayako Bun, Masayuki Nagahashi, Mamiko Kuroiwa, Miki Komatsu, Yasuo Miyoshi

**Affiliations:** Department of Surgery, Division of Breast and Endocrine Surgery, School of Medicine, Hyogo Medical University, 1-1 Mukogawa-cho, Nishinomiya, Hyogo 663-8501 Japan

**Keywords:** Advanced breast cancer, Eribulin, Interleukin-6, Prognostic factor, Tumor immune microenvironment, Myeloid-derived suppressor cell

## Abstract

**Purpose:**

Eribulin is a unique anti-cancer drug which can improve overall survival (OS) of patients with metastatic breast cancer (MBC), probably by modulating the tumor immune microenvironment. The aim of this study was to investigate the clinical significance of serum levels of immune-related and inflammatory cytokines in patients treated with eribulin. Furthermore, we investigated the association between cytokines and immune cells, such as myeloid-derived suppressor cells (MDSCs) and cytotoxic and regulatory T cells, to explore how these cytokines might affect the immune microenvironment.

**Methods:**

Sixty-eight patients with MBC treated with eribulin were recruited for this retrospective study. The relationship of cytokines, including interleukin (IL)-6, to progression-free survival and OS was examined. CD4^+^ and CD8^+^ lymphocyte, MDSCs and regulatory T cell levels were determined in the blood by flow cytometry analysis.

**Results:**

In our cohort, patients with high IL-6 at baseline had shorter progression-free survival and OS compared with those with low IL-6 (*p* = 0.0017 and *p* = 0.0012, respectively). Univariable and multivariable analyses revealed that baseline IL-6 was an independent prognostic factor for OS (*p* = 0.0058). Importantly, CD8^+^ lymphocytes were significantly lower and MDSCs were significantly higher in patients with high IL-6, compared to those with low IL-6.

**Conclusion:**

Baseline IL-6 is an important prognostic factor in patients with MBC treated with eribulin. Our results show that high IL-6 is associated with higher levels of MDSCs which suppress anti-tumor immunity, such as CD8^+^ cells. It appears that eribulin is not particularly effective in patients with high IL-6 due to a poor tumor immune microenvironment.

**Supplementary Information:**

The online version contains supplementary material available at 10.1007/s10549-023-07086-9.

## Introduction

Metastatic and recurrent breast cancers tend to become refractory to chemotherapies, and a limited number of agents have been shown to further extend overall survival (OS) after treatment with major chemotherapeutic agents, such as taxanes and anthracyclines [1–5]. Eribulin mesylate, which is a unique inhibitor of microtubule dynamics, is one of the anti-cancer drugs that can extend the OS of patients with metastatic breast cancer (MBC) who have received at least two prior chemotherapy regimens for late-stage disease [6–11]. Interestingly, eribulin extends OS without extending progression-free survival (PFS) of patients with MBC [8]. This indicates that the effect of eribulin on the tumor microenvironment, such as suppression of epithelial-mesenchymal transition and improvement of the hypoxic microenvironment by vascular normalization, may affect treatment results after eribulin [12, 13]. Moreover, in the phase III EMBRACE trial, we identified that high absolute lymphocyte count (ALC) at baseline is significantly associated with longer OS in eribulin-treated patients, but not in patients treated with the physician’s choice, which strongly suggests an association between eribulin efficacy and immune response [14–16].

Although there is strong evidence that eribulin has a role in the tumor immune microenvironment to enhance anti-tumor immunity, the precise mechanisms involved in patients undergoing eribulin treatment are unknown. This is due to the difficulty of directly monitoring the tumor microenvironment in the daily clinical setting, which is currently only possible with repeated biopsies of the metastatic tumors. In addition, the ALC and neutrophil-to-lymphocyte ratio (NLR), which are related to the effects of eribulin [14, 15, 17], reflect the immune status of the whole body, and these parameters may not directly reflect the tumor immune microenvironment. Therefore, there is a paucity of data examining the actual tumor immune microenvironment in patients with MBC treated with eribulin.

Cytokines are involved in regulating the tumor microenvironment and can be measured in the blood [18, 19], potentially helping to monitor the tumor microenvironment in daily practice. Indeed, some cytokines are associated with the actions of eribulin, and may reflect changes in the tumor immune microenvironment. In this study, we focused on interleukin (IL)-6, soluble IL-2 receptor (sIL-2R) and tumor necrosis factor (TNF)-α as cytokines related to tumor immunity and microenvironment [20–23]. Furthermore, immunosuppressive immune cells, such as myeloid-derived suppressor cells (MDSCs), regulatory T cells (Tregs) and cytotoxic CD8^+^ T cells play important roles to suppress anti-tumor immunity in the tumor microenvironment. Since MDSCs and Tregs are present both in the tumor microenvironment and in the blood, their role can be inferred from blood tests. The aim of this study was to investigate the clinical significance of serum levels of the immune and inflammatory cytokines, IL-6, sIL2-R and TNF-α, in patients with MBC treated with eribulin. Furthermore, we investigated the association between cytokines and immune cells, such as MDSCs and cytotoxic and regulatory T cells, in the blood of these patients to explore the effect of these cytokines on the immune microenvironment in patients treated with eribulin.

## Materials and methods

### Patient eligibility

A total of 68 patients with MBC treated with eribulin at Hyogo Medical University Hospital from December 2014 to March 2023 were recruited for this retrospective study. Patients were eligible if they had received more than one cycle of eribulin therapy. All participants were confirmed to have primary breast cancer through histologic examination, and a locally-advanced stage or metastasis was confirmed through diagnostic radiography using computed tomography, whole-body bone scintigraphy, or 2-([18]F)-fluoro-2-deoxy-D-glucose positron emission tomography.

All patients were classified by the combination of hormone receptor and human epidermal growth factor 2 (HER2) expression. Hormone receptor was considered negative if there were less than 1% positive tumor nuclei in the sample on immunohistochemical testing, in the presence of expected reactivity of internal (normal epithelial elements) and external controls. HER2-negative was defined as either an immunohistochemistry score of zero/1+ or 2+ with no HER2 amplification, as confirmed by in situ hybridization.

### Determination of the NLR, ALC and cytokine levels in peripheral blood

Baseline values for ALC, NLR and cytokine levels, including IL-6, sIL-2R and TNF-α, were determined in peripheral blood before the day of the first treatment with eribulin. The neutrophil and lymphocyte counts were measured automatically using a Sysmex XN-9000 or XN-1000 hematology analyzer (Sysmex Corp, Kobe, Japan). NLR was defined as the neutrophil count divided by the lymphocyte count. Serum samples were sent to an external clinical laboratory (SLR, Inc. Tokyo, Japan) to determine IL-6, sIL-2R and TNF-α levels. IL-6 and sIL-2R were measured by chemiluminescent enzyme immunoassay (Fujirebio, Inc, Tokyo, Japan) and TNF-α was measured by enzyme-linked immunosorbent assay (R&D Systems, Minneapolis, US).

### Flow cytometry analysis

For research purposes, we routinely perform flow cytometry analysis on fresh blood from patients undergoing chemotherapy if informed consent is obtained from the patient. Peripheral blood was collected in a tube and diluted with D-PBS (−) (Nacalai Tesque Inc., Kyoto, Japan) and layered onto Ficoll–Paque PLUS (Cytiva, Marlborough, US) using SepMate-50 tubes (STEM CELL Technologies, Vancouver, Canada). The tubes were centrifuged at 1200×*g* for 20 min at 20 °C. The top layer containing the enriched mononuclear cells was poured off and collected in another tube. The tubes were then centrifuged at 300×*g* for 10 min at 20 °C prior to washing. 1× RBC Lysis Buffer (Thermo Fisher Scientific, Waltham, US) was added to the cell pellet containing red blood cells, then the cells were washed twice with D-PBS (−). Cells were counted using a Countess 2 FL Automated Cell Counter (Thermo Fisher Scientific) and used for flow cytometry analysis.

Cells were incubated with human serum AB (GemCell, Seven Hills, Australia) for 30 min at 4 °C in the dark for blocking, then stained with conjugated monoclonal antibodies (mAbs) for 30 min at 4 °C in the dark. After staining, cells were washed with Cell Staining Buffer (BioLegend, San Diego, US), and then fixed with True-Nuclear 1× Fix Concentrate (BioLegend) for 15 min at room temperature in the dark for cell surface staining and for 45 min at room temperature for intracellular staining. After cell surface staining, cells for intracellular staining were stained with conjugated mAbs suspended in True-Nuclear 1× Perm Buffer (BioLegend) for 30 min. Stained cells were detected by a BD LSRFortessa X-20 cell analyzer (BD Biosciences, San Jose, US) and analyzed with BD FACSDiva software.

### Immune phenotypic profiles

Tregs were defined as CD4^+^CD25^+^FoxP3^+^ cells, while MDSCs were defined as CD11b^+^CD14^+^CD33^+^ cells, according to previous studies [24–26]. The following immune cell subsets were analyzed using flow cytometry and the listed Abs. T-cell subsets: FITC-conjugated anti-CD4 mAb (RPA-T4, BioLegend); PE-conjugated anti-CD8α mAb (HT8a, BioLegend); and APC-conjugated with anti-CD3 mAb (HT3a, BioLegend). Tregs: Alexa Flour 488-conjugated anti-FoxP3 mAb (Clone: 259D, BioLegend); PE-conjugated anti-CD25 mAb (M-A251, BioLegend); and APC-conjugated anti-CD4 mAb (RPA-T4, BioLegend). MDSCs: FITC-conjugated with anti-CD14 mAb (63D3, BioLegend); PE-conjugated with anti-CD33 mAb (WM53, BioLegend); and APC-conjugated with anti-CD11b mAb (M1/70, BioLegend). Alexa Flour 488-conjugated with anti-mouse IgGk (MOPC-21, BioLegend) and PE-conjugated anti-mouse IgGk mAb (MOPC-21, BioLegend) were used for isotype controls.

### Statistical analysis

ALC, NLR and each cytokine were classified into low and high groups, based on cut-off values for each factor, and the PFS and OS between low and high groups were compared by Kaplan–Meier plots. Univariable and multivariable analyses of clinicopathological factors contributing to OS prolongation were performed using the Cox proportional-hazards model to obtain hazard ratios and 95% confidence intervals. The relationships between the clinicopathologic characteristics and IL-6 were evaluated using the χ^2^ or Fisher’s exact test, as appropriate. *p* < 0.05 was considered to indicated a significant difference. All statistical analyses were performed using JMP Pro Version 15.

## Results

### Determination of optimal cut-off values for ALC, NLR, IL-6, sIL-2R and TNF-α for OS

The cut-off values for NLR and ALC were set at 3 and 1500/µL, respectively, in accordance with previous studies [14]. Based on the receiver operating characteristic curve calculated using the Youden index for area under the curve (AUC), the optimal cut-off values for OS were determined as 3.4 pg/mL for IL-6 (AUC, 0.734; sensitivity, 0.856; specificity, 0.621); 403 U/mL for sIL-2R (AUC, 0.731; sensitivity, 0.771; specificity, 0.634) and 0.73 pg/mL for TNF-*α* (AUC, 0.517; sensitivity, 0.289; specificity, 0.832).

### PFS and OS of patients according to baseline levels of IL-6, sIL-2R and TNF-α

**Fig. 1:**
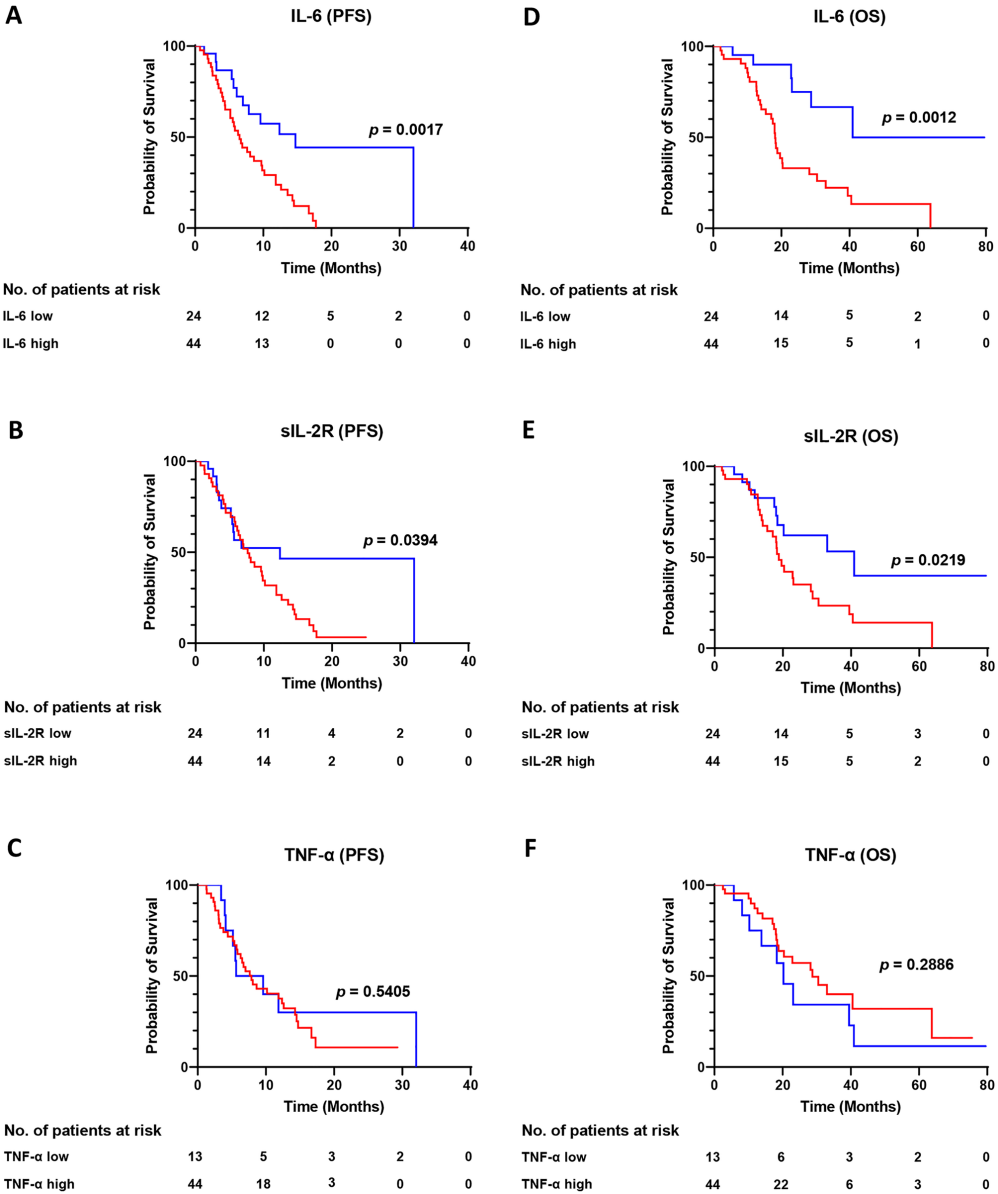
Kaplan–Meier plots of progression-free survival (PFS) (**A**–**C**) and overall survival (OS) (**D**–**F**) in 68 patients treated with eribulin according to baseline levels of interleukin (IL)-6 (**A**, **D**), soluble IL-2 receptor (sIL-2R) (**B**, **E**) and tumor necrosis factor (TNF)-α (**C**, **F**). Blue and red lines show low and high levels, respectively

First, we assessed the PFS and OS of the 68 patients treated with eribulin according to baseline levels of IL-6, sIL-2R and TNF-α (Fig. 1). Patients with high baseline IL-6 levels showed significantly shorter PFS than those with low baseline IL-6 levels (*p* = 0.0017, Fig. 1A). Patients with high sIL-2R at baseline also showed significantly poorer PFS than those with low sIL-2R (*p* = 0.0394, Fig. 1B). Baseline TNF-α levels were not associated with PFS (*p* = 0.5405, Fig. 1C). Patients with high baseline IL-6 had poorer OS, compared with those with low IL-6 at baseline, and interestingly, the difference was even greater than with PFS (*p* = 0.0012, Fig. 1D). Patients with high sIL-2R at baseline also showed significantly shorter OS than those with low sIL-2R (*p* = 0.0219, Fig. 1E). Baseline TNF-α levels were not associated with OS (*p* = 0.2886, Fig. 1F).

### Univariable and multivariable analyses for OS in patients treated with eribulin

Next, we performed univariable and multivariable analyses for OS in patients treated with eribulin to assess the clinical impact of baseline IL-6, sIL-2R and TNF-α levels (Table 1). Univariable analysis revealed that IL-6 and sIL-2R levels were significant prognostic factors for OS (*p* = 0.0026 and *p* = 0.0258, respectively, Table 1). Multivariable analysis, including IL-6 and other clinical parameters, revealed that IL-6 level was an independent prognostic factor for OS (*p* = 0.0058, Table 1). However, multivariable analysis, including sIL-2R and other clinical parameters, revealed that sIL-2R level was not an independent prognostic factor for OS (*p* = 0.2827, Table 1).

**Table 1:** Univariable and multivariable analyses of overall survival for patients treated with eribulin

	Univariable analysis		Multivariable analysis			
	HR (95% CI)	*p-*value	HR (95% CI)^a^	*p*-value^a^	HR (95% CI)^b^	*p-*value^b^
Menopausal status						
Premenopausal	1		1		1	
Postmenopausal	2.385 (0.920–6.166)	0.0487	1.216 (0.432–3.423)	0.7111	1.536 (0.549–4.301)	0.4138
Subtype						
HR+HER2−	1		1		1	
HR-HER2−	1.551 (0.692–3.475)	0.2864	2.999 (1.099–8.189)	0.0320	1.498 (0.658–3.413)	0.3558
HER2+	0.793 (0.323–1.9444)	0.6124	1.225 (0.464–3.237)	0.6819	0.892 (0.336–2.367)	0.8180
Advanced/recurrence						
Advanced	1		1		1	
Recurrence	1.720 (0.714–4.144)	0.2267	1.158 (0.455–2.950)	0.7584	1.547 (0.613–3.906)	0.3556
Site of disease						
Non-visceral	1		1		1	
Visceral	0.534 (0.274–1.039)	0.0649	0.539 (0.255–1.140)	0.1060	0.603 (0.292–1.247)	0.1724
Treatment lines						
1 line	1		1		1	
>2 lines	1.551 (0.792–3.036)	0.2006	0.983 (0.471–2.049)	0.9629	1.201 (0.584–2.467)	0.6188
NLR baseline						
Low	1					
High	1.647 (0.858-3.200)	0.1343				
ALC baseline						
Low	1					
High	0.773 (0.373–1.514)	0.4679				
IL-6 baseline						
Low	1		1			
High	3.849 (1.599–9.266)	0.0026	4.517 (1.548–13.185)	0.0058		
sIL-2R baseline						
Low	1				1	
High	2.286 (1.105–4.730)	0.0258			1.592 (0.682–3.717)	0.2827
TNF-*α* baseline						
Low	1					
High	0.655 (0.299–1.438)	0.2921				

*HR* hazard ratio; *CI* confidence interval; *HR* hormone receptor; *HER2* human epidermal growth factor 2; *NLR* neutrophil-to-lymphocyte ratio; *ALC* absolute lymphocyte count; *IL* interleukin; *sIL-2R* soluble IL-2 receptor; *TNF* tumor necrosis factor

^a^Data were obtained by multivariable analysis including baseline IL-6 and other clinical parameters

^b^Data were obtained by multivariable analysis including baseline sIL-2R and other clinical parameters

### Clinical impact of IL-6 levels at baseline and after treatment

**Fig. 2:**
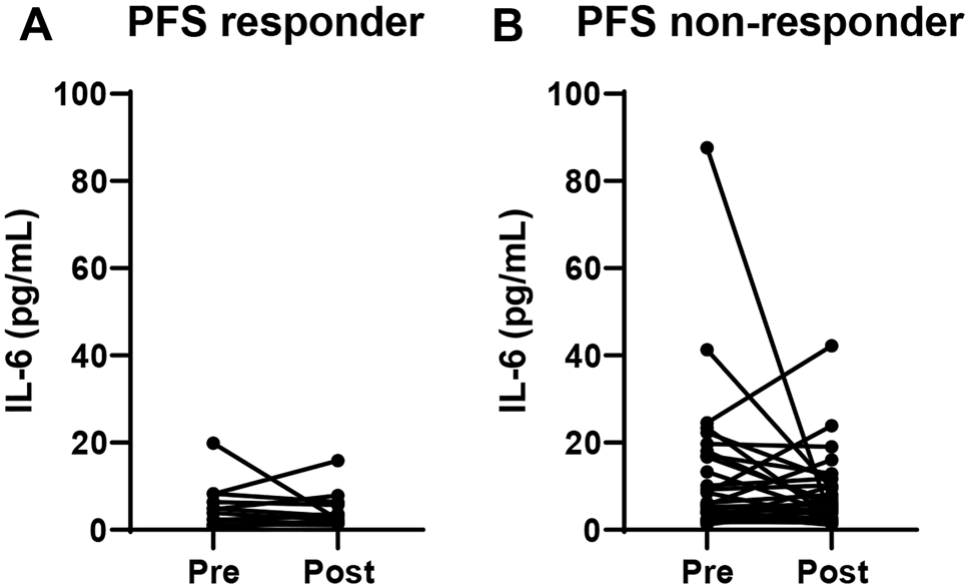
Interleukin (IL)-6 levels at baseline (Pre) and after treatment (Post) in responders (progression-free survival [PFS] ≥ 12 months) (A) and non-responders (PFS < 12 months) (B)

Since IL-6 levels at baseline were significantly associated with the clinical outcomes of patients treated with eribulin, we next investigated which indicator was the most important among IL-6 levels: baseline, post-treatment, or changes in IL-6 between baseline and post-treatment. To this end, we assessed the PFS and OS of the 68 patients treated with eribulin according to their IL-6 levels after the first course of treatment with eribulin (Supplementary Fig. 1). Although post-treatment IL-6 levels were significantly associated with PFS (*p* = 0.0374, Supplementary Fig. 1A), post-treatment IL-6 levels were not significantly associated with OS (Supplementary Fig. 1B) in these patients. We next compared IL-6 levels at baseline and after the first treatment cycle in responders (PFS ≥ 12 months) and non-responders (PFS < 12 months) to assess the impact of changes in IL-6 before and after treatment. Most of the responders showed low IL-6 levels, both at baseline and at post-treatment (Fig. 2A). In contrast, most of the patients with high levels of IL-6, either at pre- or post-treatment, were non-responders (Fig. 2B).

### Clinicopathological characteristics of patients with high IL-6 levels

Although our results indicate the importance of baseline IL-6 level as a prognostic indicator, we do not measure IL-6 levels in daily practice. Therefore, we examined the association of IL-6 level and other clinicopathological characteristics to reveal the characteristics of patients with high IL-6 levels at baseline. Univariable analysis revealed that patients with high baseline IL-6 had significantly lower albumin levels (*p* = 0.0007), higher C-reactive protein levels (*p* < 0.0001), higher modified Glasgow prognostic scores (*p* = 0.0064), lower prognostic nutritional indices (*p* = 0.0040), and lower platelet–lymphocyte ratios (*p* = 0.0361) than patients with low baseline IL-6 levels. Multivariable analysis revealed that patients with high baseline IL-6 had higher C-reactive protein levels (*p* = 0.0016), and lower prognostic nutritional indices (*p* = 0.0107) than patients with low baseline IL-6 levels.(Supplementary Table 1).

### Anti-tumor immunity associated with IL-6 levels in patients treated with eribulin

**Fig. 3:**
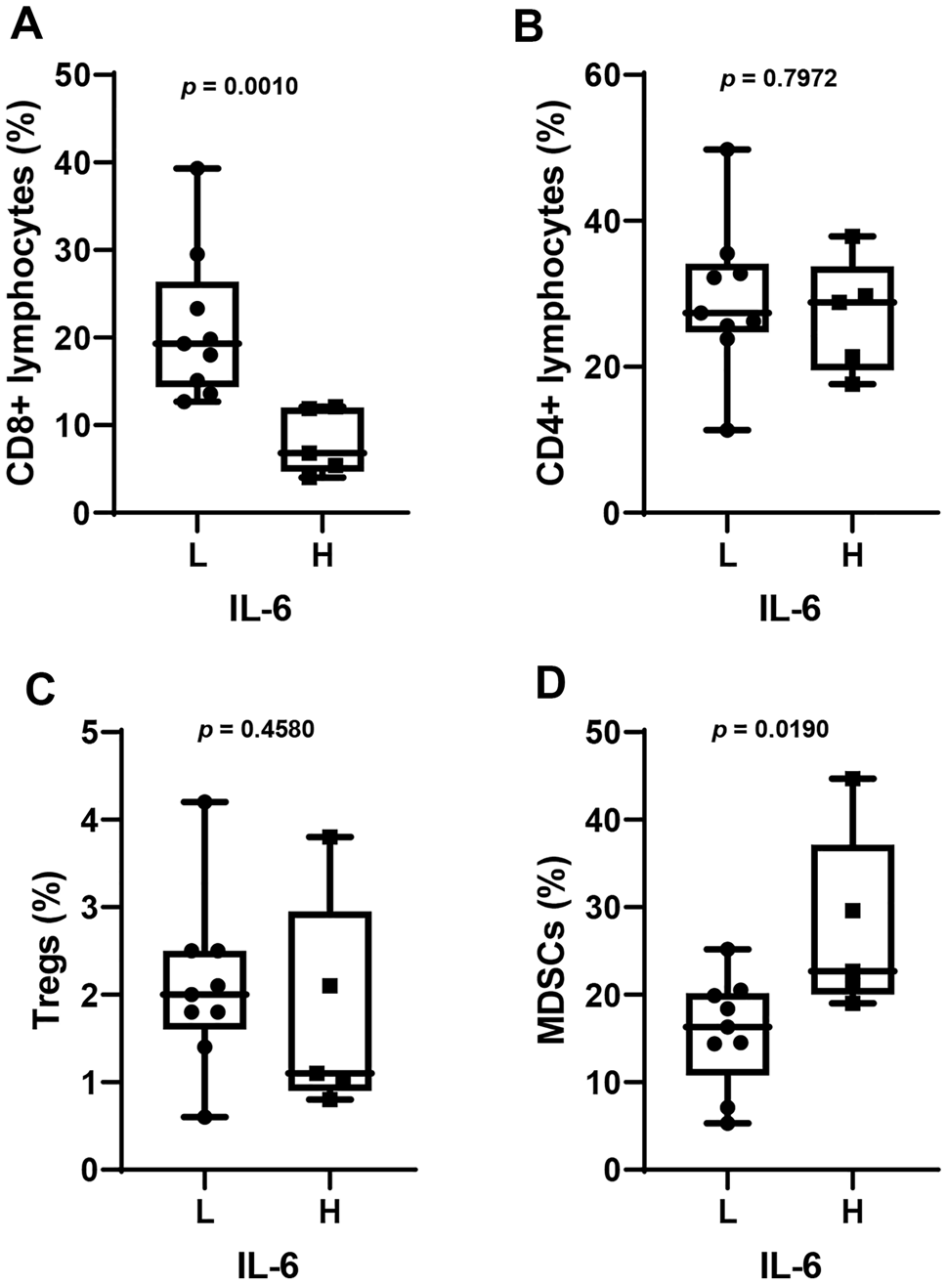
Proportions of peripheral blood lymphocyte fractions (CD8^+^ (A) and CD4^+^ (B) lymphocytes), regulatory T cells (Tregs) (C) and myeloid-derived suppressor cells (MDSCs) (D) in patients with low (L) and high (H) interleukin (IL)-6 at baseline. The cut-off value for IL-6 was set at 3.4 pg/mL

We further explored the mechanisms underlying the poorer prognosis in patients with high IL-6 levels. We hypothesized that IL-6 levels might be associated with the state of anti-tumor immunity related to the treatment effect of eribulin, and so we analyzed the peripheral blood fractions for helper (CD4^+^) and cytotoxic (CD8^+^) lymphocytes, Tregs and MDSCs involved in tumor immunity at baseline. Interestingly, CD8^+^ lymphocytes, but not CD4^+^ lymphocytes, were significantly lower in patients with high IL-6, compared with those with low IL-6 (*p* = 0.0010 and *p* = 0.7972, respectively; Fig. 3A, B). Moreover, MDSCs were significantly higher in patients with high IL-6, compared with low IL-6 (*p* = 0.0190), although Tregs did not show any significant difference between the two patient groups (Fig. 3C, D).

## Discussion

Eribulin is a distinctive anti-cancer drug which improves OS without extending PFS in patients with MBC [8]. ALC is a predictive marker for MBC patients treated with eribulin [14–16], and eribulin plays a role in regulating the tumor immune microenvironment [12, 13]; however, the underlying mechanisms of this regulatory role are not fully understood. In this study, we demonstrated that baseline IL-6 level is an independent prognostic factor for OS in patients with MBC treated with eribulin. Moreover, patients with high IL-6 levels had a low proportion of CD8^+^ cells and higher MDSC levels. These findings suggest the importance of an IL-6-associated immune mechanism underlying the effects of eribulin in patients with MBC.

Our current findings are in line with a previous study which showed that high IL-6 and IL-8 levels are significantly correlated with poorer survival of MBC patients treated with eribulin [27]. Transforming growth factor (TGF)-β is also reported to be an independent prognostic factor for MBC [28]. Basic research has shown that IL-6 and TGF-β closely interact with each other in the breast cancer microenvironment [29, 30], so both of these mediators may be associated with prognosis in patients receiving eribulin. Taken together, these results suggest that IL-6 is one of the major factors that predicts a poorer prognosis for patients with MBC treated with eribulin.

Our data also revealed that high IL-6 levels in MBC patients are associated with a low proportion of CD8^+^ lymphocytes, which play a central role in anti-tumor immunity [31, 32]. We also showed that high IL-6 was associated with high MDSC levels. It is well known that MDSCs suppress anti-tumor immunity, such as by inhibiting lymphocyte function, including CD8^+^ lymphocytes [33, 34]. Our findings indicate that a high level of IL-6 in MBC patients is associated with a pro-inflammatory tumor microenvironment, which promotes recruitment of MDSCs and suppresses anti-tumor immunity, including CD8^+^ lymphocyte activity.

In this study, baseline IL-6 levels reflected the clinical outcome of patients treated with eribulin, more so than post-treatment IL-6 levels, or changes in IL-6 levels before and after treatment. Others have also reported that baseline IL-6 levels are associated with the prognosis of patients treated with eribulin [27]. Furthermore, changes in TGF-β after treatment are also an important prognostic factor for MBC patients treated with eribulin [27]. It is possible that the difference in clinical significance of each cytokine reflects the difference in the role of each cytokine in the dynamic changes in tumor immunity. Moreover, in MBC patients, responders (non-progressive disease cases) to eribulin treatment have higher ALC at baseline, compared with non-responders, and the responders show further increases in ALC after treatment [35]. Taken together, these findings suggest that responders to eribulin have a favorable initial immune microenvironment, and that the immune microenvironment may be further improved with eribulin treatment. Considering that patients with low IL-6 had fewer MDSCs and higher CD8^+^ cells in our study, it is likely that a favorable immune microenvironment with low IL-6 level at baseline is especially important for the efficacy of eribulin.

Our data suggest that not only IL-6, but also immune cells, such as MDSCs and CD8^+^ cells, in peripheral blood are associated with the tumor microenvironment and patient outcome. Peripheral blood immune cell subsets, including NLR, have been reported to reflect the tumor microenvironment and anti-tumor immune response, and ultimately clinical outcome [36]. We have also reported that NLR and/or ALC reflect the outcome of breast cancer patients with some specific treatments [37–39]. These findings indicate that peripheral blood immune cells may reflect the tumor microenvironment of breast cancer patients, which is related to patient outcome.

This study has limitations. First, it was retrospective in nature with a comparatively small number of patients. Second, we did not measure other important cytokines, such as TGF-β. However, our data revealed important findings, including the clinical importance of IL-6 level at baseline as a prognostic marker for MBC patients treated with eribulin. Of note, as far as we know, this is the first study to reveal the involvement of MDSCs in the immune functions associated with IL-6 in eribulin treatment. Therefore, we believe that our data are valuable for understanding the actions of eribulin in the tumor immune microenvironment.

In conclusion, we suggest that the baseline IL-6 level is an important prognostic factor in patients with MBC treated with eribulin. Since high IL-6 induces MDSCs and suppresses anti-tumor immunity, reflected by reduced CD8^+^ lymphocyte counts, it is possible that eribulin is not sufficiently effective in patients with high IL-6 levels due to a poor tumor immune microenvironment.

### Supplementary Information

Below is the link to the electronic supplementary material.

Supplementary material 1 (PDF 213.9 kb)

## Data Availability

The data are not publicly available, and will be shared on reasonable request to the corresponding author.
