# Supplementary material for: Baseline interleukin-6 is a prognostic factor for patients with metastatic breast cancer treated with eribulin
Source: Breast Cancer Res Treat. 2023 Sep 21;202(3):575–83. doi: 10.1007/s10549-023-07086-9 (PMC10564839; doi:10.1007/s10549-023-07086-9)
Supplement: Supplementary file 1 — Supplementary material 1 (PDF 213.9 kb) [file 10549_2023_7086_MOESM1_ESM.pdf]

Supplementary Figure 1

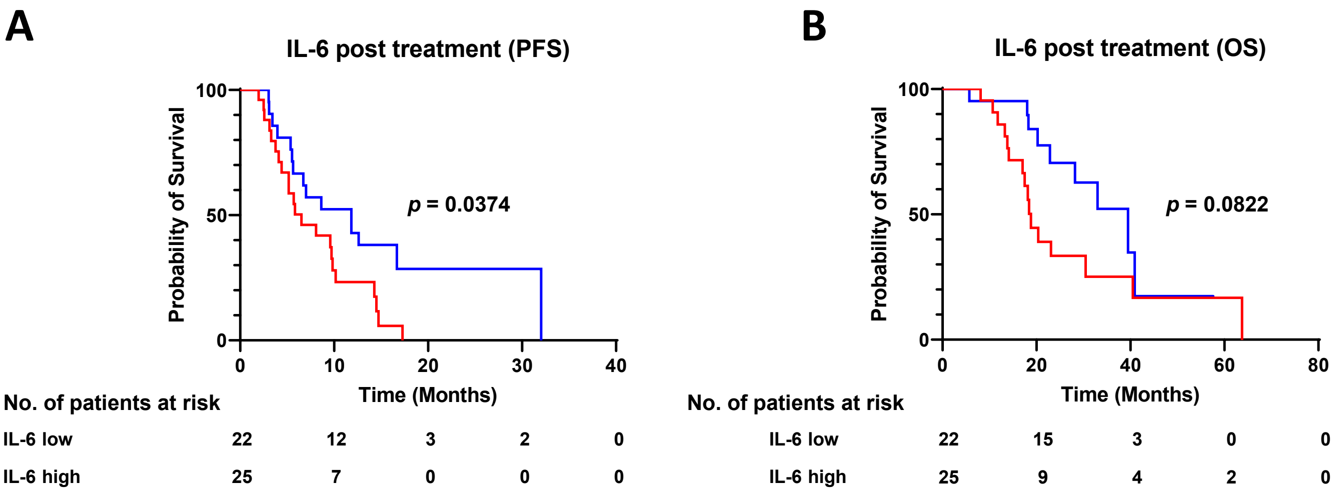

Supplementary Fig. 1 Kaplan-Meier plots of progression-free survival (PFS) (A) and overall survival (OS) (B) in 68 patients treated with eribulin according to interleukin (IL)-6 levels after the first course of treatment. The cut-off value for IL-6 was set at 3.4 pg/mL.

Supplementary Table 1 Clinicopathological characteristics of patients based on baseline IL-6

	No. of patients (%)			Univariable analysis	Multivariable analysis
	Total (N = 68)	IL-6 low (N = 24)	IL-6 high (N = 44)	*p*-value	*p*-value
Menopausal				0.2070	0.7823
Pre-	12 (18)	6 (9)	6 (9)		
Post-	55 (81)	17 (25)	38 (56)		
Unknown	1 (1)	1 (1)	0 (0)		
Subtype				0.6342	0.2185
Luminal	42 (62)	13 (19)	29 (43)		
HER2	12 (17)	5 (7)	7 (10)		
Triple negative	14 (21)	6 (9)	8 (12)		
Advanced/recurrence				0.8328	0.1569
Advanced	16 (24)	6 (9)	10 (15)		
Recurrence	52 (76)	18 (26)	34 (50)		
Site of disease				0.2942	0.5463
Visceral	31 (45)	13 (19)	18 (26)		
Non-visceral	37 (55)	11 (16)	26 (39)		
Treatment line				0.1079	0.9700
1	28 (41)	13 (19)	15 (22)		
≧2	40 (59)	11 (16)	29 (43)		
NLR				0.0940	0.6265
Low	36 (53)	16 (24)	20 (29)		
High	32 (47)	8 (12)	24 (35)		
ALC				0.7466	0.3256
Low	47 (69)	16 (24)	31 (45)		
High	21 (31)	8 (12)	13 (19)		
Albumin*				0.0007	0.1249
Low (≦3.5 g/dL)	27 (40)	3 (5)	24 (35)		
High (＞3.5 g/dL)	41 (60)	21 (31)	20 (29)		
CRP*				< 0.0001	0.0016
Low (≦0.3 mg/dL)	34 (50)	21 (31)	13 (19)		
High (＞0.3 mg/dL)	33 (49)	3 (5)	30 (44)		
Unknown	1 (1)	0 (0)	1 (1)		
mGPS				0.0064	0.6710
0	49 (72)	23 (34)	26 (38)		
1	8 (12)	1 (1)	7 (11)		
2	10 (15)	0 (0)	10 (15)		
Unknown	1 (1)	0 (0)	1 (1)		
PNI†				0.0040	0.0107
Low (≦47.8)	53 (78)	14 (21)	39 (57)		
High (＞47.8)	15 (22)	10 (15)	5 (7)		
PLR†				0.0361	0.5195
Low (≦272)	49 (72)	21 (31)	28 (41)		
High (＞272)	19 (28)	3 (5)	16 (23)		

*Based on cut-off values for normal clinical laboratory values. †Based on optimal cut-off values for overall survival determined by the receiver operating characteristic curve calculated using the Youden index for area under the curve. *IL* interleukin; *HER2* human epidermal growth factor 2; *NLR* neutrophil-to-lymphocyte ratio; *ALC* absolute lymphocyte count; *CRP* C-reactive protein; *mGPS* modified Glasgow prognostic score; *PNI* prognostic nutritional index; *PLR* platelet-lymphocyte ratio
